# Comparative Rheological Characterization of Hyaluronic Acid Viscosupplements for Synovial Fluid Restoration in Osteoarthritis and Knee Biomechanics

**DOI:** 10.3390/gels12100882

**Published:** 2026-09-30

**Authors:** Alejandro Melero, Ander Pino, José María Alonso, Nagore Martínez de Cestafe, Claudia Goenaga Ibeas, Amaia Huguet-Casquero, Miguel Ucelay López de Heredia, Raúl Pérez González

**Affiliations:** i+Med S. Coop., Alava Technology Park, C/Hermanos Elhuyar 6, 01510 Vitoria-Gasteiz, Spain; alejandro.melero@imasmed.com (A.M.); ander.pino@imasmed.com (A.P.); nmartinez@imasmed.com (N.M.d.C.); cgoenaga@imasmed.com (C.G.I.); amaia.huguet@imasmed.com (A.H.-C.); miguel.ucelay@imasmed.com (M.U.L.d.H.); rperez@imasmed.com (R.P.G.)

**Keywords:** hyaluronic acid, viscosupplementation, rheology, synovial fluid, osteoarthritis, knee osteoarthritis, viscoelasticity, normal stress difference

## Abstract

Synovial fluid (SF) rheological properties deteriorate progressively in knee osteoarthritis (OA) due to a reduction in hyaluronic acid (HA) concentration and molecular weight, compromising joint lubrication and load distribution. Restoring these properties through intra-articular viscosupplementation with HA is a widely used therapeutic strategy. However, commercially available linear HA formulations differ substantially in their mechanical behavior, and single-parameter assessments are insufficient for capturing their functional differences. In this study, eight commercially available intra-articular linear HA hydrogels—TrHLINE 1%^®^, TrHLINE 2%^®^, Hyalubrix^®^, Hyalgan Bio^®^, Pronolis^®^ HD 2.5%, Biolevox™ HA ONE 2.5%, Suplasyn^®^, and Arthrum H 2%^®^—were evaluated under standardized conditions using steady-state viscosity, mechanical stress response, first normal stress difference (N1), storage and loss moduli (G′, G″), loss factor (tan δ), and elasticity percentage. Results revealed three distinct functional profiles: lubricant-oriented with low to moderate structural organization (Hyalgan Bio^®^, Suplasyn^®^, TrHLINE 1%^®^); balanced viscoelastic with sustained structural persistence (TrHLINE 2%^®^, Hyalubrix^®^, Arthrum H 2%^®^); and high initial rigidity with reduced dynamic persistence (Biolevox™ HA ONE 2.5%, Pronolis^®^ HD 2.5%). These findings demonstrate that multi-parametric rheological benchmarking effectively discriminates functional differences among HA viscosupplements and supports indication-oriented product selection based on OA severity and patient mechanical demand.

## 1. Introduction

Osteoarthritis (OA) is the most prevalent musculoskeletal disorder worldwide, affecting approximately 607 million people as of 2021—equivalent to 7.7% of the global population. The total number of cases is projected to continue rising through 2035, primarily driven by population ageing and rising obesity rates [1]. The disease is characterized by progressive articular cartilage deterioration, osteophyte formation, subchondral bone remodeling, and chronic synovial membrane inflammation [2,3]. Knee OA (KOA) in particular leads to joint pain, increased joint stiffness—clinically manifesting as reduced range of motion and greater resistance to passive movement—motion limitation, fatigue, impaired sleep, and reduced quality of life [4,5]. Beyond age, sex, and body mass index, repeated joint stress and traumatic injury—common in both recreational and competitive physical activity—represent well-established risk factors for KOA onset and progression [6,7,8]. Therefore KOA is highly relevant to sports medicine and traumatology.

Within the healthy knee, synovial fluid (SF) fulfils critical biomechanical functions: it provides lubrication to articular surfaces, distributes compressive loads, dissipates mechanical energy, and protects cartilage from accelerated wear [9,10,11]. These properties arise primarily from hyaluronic acid (HA), a high-molecular-weight glycosaminoglycan present in SF at concentrations of 1–4 mg/mL and molecular weights of 6–8 MDa in healthy joints, acting in concert with lubricin and other boundary lubricants [9,12,13,14]. Healthy SF exhibits non-Newtonian, shear-thinning behavior, with viscosity at 1 s^−1^ approximating 3 Pa·s, dropping to 0.02–0.05 Pa·s at 1000 s^−1^. The SF storage (G′) and loss (G″) moduli reach approximately 23 Pa and 7 Pa respectively, with a loss factor (tan δ) below unity, approximately 0.30, reflecting a balanced viscoelastic state [13,15,16,17]. These reference values, compiled by Fam et al. [13] from multiple original sources, derive in part from Balazs et al. [16], who characterized healthy SF from 503 knees of 268 normal male adults aged 21–45 years. As is characteristic of viscoelastic biological fluids, both G′ and G″ in healthy SF increase with angular frequency. At low frequencies, G″ exceeds G′, reflecting viscous-dominant behavior suited to slow, quasi-static loading; as frequency increases, G′ rises more steeply and eventually surpasses G″ at the crossover point, conferring elastic-dominant behavior under dynamic demand [13]. This rheological profile allows SF to behave as a lubricant under the low shear rates of slow movement (0.01–1 s^−1^) and as a more elastic, energy-absorbing medium under the high shear rates associated with demanding physical activity (10^3^–10^5^ s^−1^) [18].

In OA, SF undergoes progressive qualitative and quantitative degradation: a quantitative decline in HA concentration, and a qualitative change in HA composition and structure—namely, reduced molecular weight and chain integrity—driven mechanistically by enzymatic hydrolysis and oxidative cleavage (detailed below). HA concentration falls to 1–2 mg/mL and mean molecular weight decreases substantially, from the 6–8 MDa range characteristic of healthy SF to approximately 0.5–3.5 MDa in OA. Such molecular weight reduction is driven by enzymatic hydrolysis and oxidative cleavage of the HA backbone by reactive oxygen species (ROS) generated during synovial inflammation [9,13,19]. The resulting SF displays diminished viscosity (0.1–2 Pa·s at 1 s^−1^) and reduced storage modulus (G′ ≈ 3–7 Pa at physiological frequencies, roughly one-third of healthy SF values [13,18]). Moreover, SF loses its elastic dominance at functional loading frequencies and exhibits impaired shear-thinning capacity [13,16,18]. Critically, in a prospective cohort study, a shift in HA molecular weight distribution toward lower values was associated with greater baseline pain, and—independently of age, gender, BMI, and baseline radiographic grade—with significantly higher odds of radiographic OA progression over three years [20]. These changes translate into inadequate joint lubrication, reduced shock absorption, and progressive cartilage damage.

Intra-articular viscosupplementation with exogenous HA—a strategy first formally introduced by Balazs and Denlinger in the early 1990s [21]—aims to restore the mechanical environment of the osteoarthritic joint by replenishing the rheological properties of degraded SF. The rationale extends beyond simple viscosity replacement: an effective viscosupplement must provide adequate low-shear viscosity for lubrication and joint residence. Furthermore, it should provide sufficient flow adaptation at high shear for dynamic mobility and an appropriate viscoelastic balance to buffer impact and redistribute loads during repetitive mechanical solicitation [22,23]. These requirements are not captured by any single rheological descriptor. Parameters such as the loss factor tan δ—which distinguishes viscous-dominant from elastic-dominant behavior—and the first normal stress difference (N1)—an in vitro measure of load-responsive lateral expansion under shear—provide mechanistic information about functional performance that viscosity alone cannot convey [18,23].

The commercial landscape of HA-based viscosupplements is highly heterogeneous. Products differ in HA concentration, molecular weight, and network architecture, and their rheological behaviors vary accordingly [14,24]. Comparative interpretation of available products has been further complicated by the absence of standardized, multi-parametric testing frameworks. Most published comparisons have relied on oscillatory rheology alone or on zero-shear viscosity without integrating stress resistance, N1, or viscoelastic transition behavior [14,23,25]. Moreover, the present study focuses specifically on linear, non-crosslinked HA formulations, a clinically important class that remains insufficiently characterized relative to crosslinked products despite wide clinical use across KOA severity grades [26,27].

Therefore, the aim of this study was to perform a standardized, multi-parametric rheological benchmarking of eight commercially available linear HA intra-articular hydrogels: TrHLINE 1%^®^, TrHLINE 2%^®^, Hyalubrix^®^, Hyalgan Bio^®^, Pronolis^®^ HD 2.5%, Biolevox™ HA ONE 2.5%, Suplasyn^®^, and Arthrum H 2%^®^. This selection covers the full range of concentrations currently represented in clinical practice. Steady-state viscosity, stress response, first normal stress difference, and oscillatory frequency sweep analyses were integrated to classify the formulations according to the biomechanical requirements of SF lubrication and replacement across OA grades [28,29].

## 2. Results and Discussion

### 2.1. Steady-State Viscosity and Shear-Thinning Behavior

The present study was designed as a standardized, multi-parametric rheological benchmarking of eight commercially available products. Measurements were performed in duplicate as an instrumental quality-control step (Supplementary Materials). All eight tested formulations exhibited non-Newtonian, shear-thinning behavior—operationally defined here as a continuous, monotonic decrease in shear viscosity (η) with increasing shear rate across the entire tested range, with no plateau or Newtonian-like region observed at any point—across the explored shear rate range (0.01–1000 s^−1^), consistent with the viscoelastic nature of HA-based hydrogels (Figure 1, Table 1, and Supplementary Materials). However, the products differed markedly in both the magnitude and the rate of viscosity reduction as a function of increasing shear.

At low shear (0.1 s^−1^), three distinct viscosity tiers were apparent. Biolevox™ HA ONE 2.5% and Pronolis^®^ HD 2.5% occupied the highest range, with viscosities of 342.47 Pa·s and 331.12 Pa·s respectively. An intermediate group comprising Arthrum H 2%^®^ (138.86 Pa·s), Hyalubrix^®^ (86.89 Pa·s), and TrHLINE 2%^®^ (68.94 Pa·s) showed substantially lower but structurally meaningful values. TrHLINE 1%^®^ (3.90 Pa·s), Suplasyn^®^ (0.99 Pa·s), and Hyalgan Bio^®^ (0.31 Pa·s) formed a third group with low initial viscosity: Suplasyn^®^ (0.99 Pa·s) and Hyalgan Bio^®^ (0.31 Pa·s) do not exceed the zero-shear viscosity ceiling reported for osteoarthritic SF (0.1–1 Pa·s [13,30]), which also bounds its viscosity at 0.1 s^−1^ from above; TrHLINE 1%^®^ (3.90 Pa·s), by contrast, exceeded that ceiling at both shear rates.

At high shear (1000 s^−1^), viscosity values narrowed substantially across all products relative to the low-shear range—from a >1000-fold spread at 0.1 s^−1^ (0.31–342.47 Pa·s) to a 5.6-fold spread at 1000 s^−1^ (0.10 Pa·s, Hyalgan Bio^®^, to 0.56 Pa·s, TrHLINE 2%^®^)—reflecting the expected shear-induced disentanglement of HA chains. The extent of shear-thinning, expressed as the η:γ ratio η(1 s^−1^)/η(1000 s^−1^), differed substantially across formulations, spanning more than two orders of magnitude (3.12–877.58; Table 1). Biolevox™ HA ONE 2.5% and Pronolis^®^ HD 2.5% exhibited the highest ratios (877.58 and 509.77, respectively), indicating a steep viscosity decay. In the case of Biolevox™ HA ONE 2.5%, this decrease brings its residual viscosity at 1000 s^−1^ (0.17 Pa·s) into the range of the 1% HA formulations. TrHLINE 2%^®^ showed a moderate and well-distributed shear-thinning ratio of 94.88, similar in order of magnitude to Hyalubrix^®^ (148.81) and Arthrum H 2%^®^ (164.82). By contrast, TrHLINE 1%^®^ (23.84), Suplasyn^®^ (6.94), and Hyalgan Bio^®^ (3.12) exhibited limited shear-thinning, consistent with more viscous, predominantly fluid-like systems. Overall, all eight formulations displayed a monotonic, continuous decrease in viscosity across the entire tested range (0.1–1000 s^−1^), with no plateau or shear-thickening observed at any point; the rate of this decline, however, varied substantially between products, spanning more than two orders of magnitude in the η:γ ratio (3.12–877.58; Table 1).

### 2.2. Mechanical Stress Response and Structural Stability

Mechanical stress (σ) as a function of shear rate revealed further functional differentiation among the formulations, particularly regarding the persistence of internal structural organization under increasing shear stress (Figure 2).

TrHLINE 2%^®^, Hyalubrix^®^, and Arthrum H 2%^®^ displayed high and continuously increasing stress profiles across the tested range, reaching values in the upper tier (~200–900 Pa at 1000 s^−1^) without evidence of structural breakdown, defined as a decline in mechanical stress (σ) following a peak value; no such decline was observed for these three formulations, whose stress profiles rose monotonically across the entire tested shear rate range. This sustained response indicates that these formulations maintain internal network coherence even under high mechanical demand. TrHLINE 1%^®^ remained within the lower-stress profile but occupied its upper end, showing greater mechanical resistance than Hyalgan Bio^®^ and Suplasyn^®^ while remaining clearly below the sustained high-resistance profiles of TrHLINE 2%^®^, Hyalubrix^®^, and Arthrum H 2%^®^. Its stress response increased without a subsequent decline across the tested shear rate range, indicating low-to-moderate mechanical resistance rather than a distinct stress-response pattern.

A qualitatively different pattern was observed for Biolevox™ HA ONE 2.5% and Pronolis^®^ HD 2.5%. Both exhibited high initial stress values—consistent with their elevated low-shear viscosity—but showed a subsequent decline at approximately 30–50 s^−1^ (Figure 2), indicating a loss of structural persistence beyond an initial resistance threshold. This behavior distinguishes them from TrHLINE 2%^®^, Hyalubrix^®^, and Arthrum H 2%^®^—the stable-resistance group described above—despite their overall higher starting values.

Hyalgan Bio^®^ and Suplasyn^®^ remained at the lower end of the stress spectrum throughout the explored range, with profiles consistent with predominantly viscous, weakly structured fluids that offer limited resistance under mechanical solicitation.

### 2.3. First Normal Stress Difference (N1)

The first normal stress difference, an in vitro rheological parameter reflecting the development of load-responsive lateral pressure under shear flow, is mechanistically related to the ability of the material itself—not the joint—to redistribute mechanical loads and showed markedly distinct patterns across the eight products (Figure 3).

Below approximately 1 s^−1^, N1 values remained near zero and were not meaningfully distinguishable between products; divergence in curve shape—specifically, the peak-and-decline profile distinguishing Biolevox™ HA ONE 2.5% and Pronolis^®^ HD 2.5% from the continuously rising profiles of the other formulations—became apparent from approximately 10–100 s^−1^ onwards, while the largest divergence in absolute N1 magnitude across products was observed at the upper end of the tested range (>500 s^−1^; Figure 3).

TrHLINE 2%^®^ and Arthrum H 2%^®^ displayed the most pronounced and sustained N1 profiles, with values rising continuously throughout the explored shear rate range and reaching approximately 2800 Pa and 2400 Pa, respectively, at the highest measured shear rates. Hyalubrix^®^ tracked closely in parallel with TrHLINE 2%^®^ and Arthrum H 2%^®^ through the low-to-moderate shear rate range, before its N1 profile flattened and declined slightly at high shear rates (Figure 3)—a comparatively modest reduction in magnitude rather than the sharp peak-and-decline pattern characteristic of Biolevox™ HA ONE 2.5% and Pronolis^®^ HD 2.5%. This sustained, non-collapsing profile—despite the high-shear plateau—places Hyalubrix^®^ in the same qualitative class as TrHLINE 2%^®^ and Arthrum H 2%^®^, representing a structurally responsive network with comparatively lower load-redistribution magnitude at the highest shear rates tested. These three formulations are distinguished by the continuous, non-reverting character of their N1 evolution.

In contrast, Biolevox™ HA ONE 2.5% and Pronolis^®^ HD 2.5% displayed an early N1 increase followed by a marked reduction beginning at approximately 10–100 s^−1^. Notably, both formulations exhibited a transient plateau—a near-constant N1 (approximately 1000–1650 Pa for Pronolis^®^ HD 2.5%, ~30–100 s^−1^; approximately 900–1050 Pa for Biolevox™ HA ONE 2.5%, ~20–100 s^−1^)—preceding the subsequent decline (Figure 3). This plateau suggests an intermediate structural stage in which the network briefly sustains its load-responsive organization before yielding under continued shear, rather than an abrupt collapse immediately following the initial peak. This non-sustained profile indicates that initial elastic organization in these formulations is not maintained under progressive mechanical demand, mirroring the reduced structural persistence observed in their stress-response data (Section 2.2).

TrHLINE 1%^®^ generated a low but detectable N1 signal, consistent with a formulation at the lower boundary of load-responsive behavior. Hyalgan Bio^®^ showed negligible N1 development, with values approaching zero or marginally negative at high shear (>500 s^−1^). Suplasyn^®^ showed a similarly negligible N1 profile throughout the tested range (0.1–1000 s^−1^), paralleling the weakly structured, viscous-dominant behavior of Hyalgan Bio^®^ rather than the low-but-detectable signal observed for TrHLINE 1%. These profiles indicate an absence of meaningful in vitro load-redistribution capacity in the most fluid-like formulations.

### 2.4. Oscillatory Viscoelasticity: Storage and Loss Moduli

Oscillatory frequency sweep experiments (0.1–63 rad/s) revealed the viscoelastic character of each formulation as a function of angular frequency, expressed through the storage modulus (G′, elastic contribution) and loss modulus (G″, viscous contribution) (Figure 4).

For Hyalgan Bio^®^ and Suplasyn^®^, G″ exceeded G′ across the entire measured frequency range, indicating predominantly viscous-dominant behavior with no gel-to-liquid crossover within the explored window (Figure 4A). TrHLINE 1%^®^ similarly exhibited G″-dominant behavior throughout the physiologically relevant frequency range [31], with a G′/G″ crossover identifiable only at approximately 30 rad/s—above the frequency associated with most physiological activities. At 10 rad/s, G′ and G″ for TrHLINE 1%^®^ were 11.61 Pa and 18.88 Pa respectively (Table 1). Hyalgan Bio^®^ (G′ = 0.43 Pa, G″ = 2.95 Pa) and Suplasyn^®^ (G′ = 2.29 Pa, G″ = 8.05 Pa) remained substantially lower, displaying profiles characteristic of weakly structured HA solutions.

Among the more structured formulations (Figure 4B), a G′/G″ crossover—the point at which elastic behavior becomes dominant—was identifiable at different frequencies. The physiologically relevant frequency range for the knee spans approximately 3–19 rad/s, corresponding to walking (~3 rad/s) through running (~16–19 rad/s) [14,16,31]. Biolevox™ HA ONE 2.5% and Pronolis^®^ HD 2.5% crossed over at lower frequencies (~1–2 rad/s), below walking frequency. This means that they display elastic-dominant behavior across the entire physiological activity range—a pattern more reminiscent of a crosslinked gel than of native SF. At 10 rad/s, G′ values for Biolevox™ HA ONE 2.5% and Pronolis^®^ HD 2.5% were 357.38 Pa and 423.96 Pa respectively, exceeding G″ (169.28 Pa and 267.10 Pa). By contrast, TrHLINE 2%^®^, Hyalubrix^®^, and Arthrum H 2%^®^ crossed over at approximately 8, 3, and 2 rad/s respectively, with G′ and G″ values at 10 rad/s of 148.64/133.58 Pa, 126.36/85.91 Pa, and 189.81/118.70 Pa. TrHLINE 2%^®^ was the only formulation to transition within the physiological window itself, while Hyalubrix^®^ crossed over at its lower boundary and Arthrum H 2%^®^ immediately below it. Importantly, a crossover at higher frequency means the formulation maintains predictable viscous-dominant behavior—rheologically consistent with favorable lubrication, though not directly measured here—across a broader range of loading conditions before transitioning to elastic dominance. This broader mechanical operating range enables progressive rheological adaptation to increasing joint loads, more closely reproducing healthy SF behavior [31].

### 2.5. Loss Factor (tan δ) and Elasticity Percentage

The loss tangent (tan δ = G″/G′), representing the ratio of viscous energy dissipation to elastic energy storage, allows a finer functional differentiation of the tested formulations (Figure 5A; Table 1).

At 1 rad/s—a frequency below walking conditions [14]—tan δ values ranged over more than two orders of magnitude. Hyalgan Bio^®^ (66.1) and Suplasyn^®^ (23.4) were strongly viscous-dominant, and TrHLINE 1%^®^ (5.7) showed a clearly viscous profile consistent with a lubricant-oriented material. Biolevox™ HA ONE 2.5% (0.99) was the only formulation at unity at this frequency, indicating that the transition to elastic dominance had already occurred. Pronolis^®^ HD 2.5% (1.37), Arthrum H 2%^®^ (1.4), and Hyalubrix^®^ (1.5) remained marginally viscous-dominant, with TrHLINE 2%^®^ (2.3) more clearly so. These values are consistent with the crossover frequencies reported in Table 1 (~1, ~2, ~2, ~3, and ~8 rad/s, respectively). While tan δ provides limited functional discrimination at sub-walking frequencies, formulation-specific differences become evident when the parameter is analyzed over the full physiological frequency range.

At 10 rad/s—approximating more demanding activity—the tan δ of TrHLINE 2%^®^ fell to 0.89, approaching unity and indicating a progressive transition toward balanced viscoelastic behavior. Hyalubrix^®^ (0.67) and Arthrum H 2%^®^ (0.62) were already below unity at this frequency, as were Pronolis^®^ HD 2.5% (0.63) and Biolevox™ HA ONE 2.5% (0.47). TrHLINE 1%^®^ (1.62) remained viscous-dominant, while Hyalgan Bio^®^ (6.84) and Suplasyn^®^ (3.50) were clearly so throughout.

The derived elasticity percentage at 10 rad/s, representing the elastic fraction of total viscoelastic energy storage, ranked products as follows: Biolevox™ HA ONE 2.5% (67.85%) > Arthrum H 2%^®^ (61.52%) > Pronolis^®^ HD 2.5% (61.34%) > Hyalubrix^®^ (59.52%) > TrHLINE 2%^®^ (52.66%) > TrHLINE 1%^®^ (38.07%) > Suplasyn^®^ (22.18%) > Hyalgan Bio^®^ (12.75%) (Figure 5B; Table 1). While Biolevox™ HA ONE 2.5% and Pronolis^®^ HD 2.5% achieved the highest maximal elasticity percentages, this must be interpreted in conjunction with their non-sustained N1 and stress-response profiles described above (Section 2.2 and Section 2.3).

### 2.6. Discussion

The results presented above demonstrate that linear HA viscosupplements are not mechanically interchangeable and that no single parameter captures their functional differences. Two SF reference profiles serve as reference throughout the following discussion: healthy SF exhibits viscosity ~3 Pa·s at 1 s^−1^ falling to 0.02–0.05 Pa·s at 1000 s^−1^, a shear-thinning ratio of 60–150, G′ ≈ 23 Pa and G″ ≈ 7 Pa at approximately 15 rad/s (10 rad/s is used herein as a fixed cross-product reference point), tan δ ≈ 0.30, and a G′/G″ crossover near 3 rad/s [13,15,18,25]. Degraded OA SF displays a viscosity of 0.1–2 Pa·s at 1 s^−1^, G′ ≈ 3–7 Pa at physiological frequencies, a viscous-dominant behavior (G″ > G′) across most of the functional loading range, and shear-thinning ratios of 5–40 [13,18,30]. These reference values are summarized in Table 2.

**The rationale for multi-parametric characterization.** Proximity to the healthy SF benchmark across this parameter set—rather than any single descriptor—is the basis for the three functional groupings that follow. Viscosity and viscoelastic moduli exhibited a near-continuous gradient across formulations. However, shear-thinning capacity, stress persistence, N1 generation, and tan δ collectively defined distinct mechanical profiles, supporting a categorical rather than ordinal classification.

The shear-thinning ratio itself falls into three non-overlapping clusters as measured [18], with clear gaps separating them: Group 1 formulations range from 3.12 to 23.84, Group 2 formulations from 94.88 to 164.82, and Group 3 formulations from 509.77 to 877.58—a nearly four-fold gap separates the highest Group 1 value from the lowest Group 2 value, and a more than three-fold gap separates the highest Group 2 value from the lowest Group 3 value. No threshold was imposed a priori; the separation is intrinsic to the measured dataset. Stress and N1 responses under ascending shear—two mechanistically related descriptors of structural persistence—partition the same eight formulations along identical lines: Group 1 formulations develop negligible-to-low resistance without a discernible growth phase; Group 2 formulations show continuously increasing, non-declining resistance across the full shear range; and Group 3 formulations show an initial increase followed by a decline, indicating a loss of structural persistence under progressive shear. The loss factor tan δ, by contrast, does not cleanly discriminate Group 2 from Group 3: at 10 rad/s, Pronolis^®^ HD 2.5% (tan δ = 0.63) falls within the range spanned by Group 2 formulations (0.62–0.89) despite belonging to Group 3 by both other criteria. This parameter is therefore treated descriptively (Table 1, Section 2.5) rather than as a classification criterion; the three functional groups are instead defined by the convergence of two independent, empirically non-overlapping descriptors—shear-thinning ratio and stress/N1 curve shape—across all eight tested products. The OA-severity and activity-level associations proposed below are rheology-derived hypotheses, and thus require prospective validation before clinical recommendation.

**Group 1—Lubricant-oriented formulations: Hyalgan Bio^®^, Suplasyn^®^, and TrHLINE 1%^®^.** Hyalgan Bio^®^ and Suplasyn^®^ behave as weakly structured viscous fluids across all tested parameters. This classification is based on rheological profile (low viscosity, viscous-dominant behavior) rather than on directly measured tribological or frictional performance; the term ‘lubricant-oriented’ is used throughout this manuscript as a rheology-based descriptor. Their viscosities at 1 s^−1^ (0.31 and 0.99 Pa·s) fall at or below degraded OA SF, and their shear-thinning ratios (3.1 and 6.9) at or below the lower bound of the OA SF range, indicating poor shear adaptation. No G′/G″ crossover was observed within the explored window; at 10 rad/s, G′ values (0.43 and 2.29 Pa) remained below the OA SF floor (G′ ≈ 3–7 Pa [13,18]), with tan δ (6.84 and 3.50) and negligible N1 confirming strongly viscous-dominant, weakly organized behavior. Both products fell at or below the OA SF rheological floor across viscosity, moduli, and N1, and would provide only limited mechanical restoration [13,18,31]. This is consistent with independent data for the originator product: Nicholls, 2018 [23] reported a comparable molecular weight (500–730 kDa) and low-shear viscosity (0.27 Pa·s) for Hyalgan^®^, with negligible shear-thinning and no measurable crossover—closely matching Hyalgan Bio^®^ (0.31 Pa·s; ratio 3.12) here.

TrHLINE 1%^®^ is mechanistically distinct within this group. Its viscosity at 1 s^−1^ (3.70 Pa·s) closely matches healthy SF (~3 Pa·s)—unlike the other two products—and its shear-thinning ratio (23.8), while below the healthy SF reference range (60–150), is the highest among the lubricant-oriented formulations. This behavior reflects a significant degree of shear-induced adaptation despite the predominantly viscous nature of the formulation. At 10 rad/s, G′ and G″ were 11.6 and 18.9 Pa (tan δ = 1.62), confirming viscous-dominant behavior; a G′/G″ crossover at approximately 30 rad/s places the elastic transition above the frequency range associated with most physiological activities, consistent with a rheological profile favorable for lubrication throughout typical loading conditions, though this has not been confirmed through tribological testing. Elasticity (38.1%) and a low but detectable N1 further distinguish it from the most fluid-like products. This formulation is the only lubricant-oriented product that meets the minimum viscosity threshold of healthy SF while retaining injectability, making it the mechanically most complete option within this group. From a clinical point of view, Group 1 aligns with early KOA (Kellgren–Lawrence grades I–II), where surface lubrication is the primary therapeutic need. Therefore, TrHLINE 1%^®^ might represent a suitable candidate within this indication.

**Group 2—Balanced viscoelastic formulations: TrHLINE 2%^®^, Hyalubrix^®^, and Arthrum H 2%^®^.** These three formulations aligned most closely with healthy SF when evaluated across the full multi-parametric profile. At 1 s^−1^, viscosities of 53.4, 52.5, and 79.1 Pa·s substantially exceeded OA SF, and their shear-thinning ratios (94.9, 148.8, and 164.8) fell within or immediately adjacent to the healthy SF reference range, with TrHLINE 2%^®^ positioned centrally within it. At 1000 s^−1^, viscosities converged to 0.35–0.56 Pa·s, retaining a residual lubricating viscosity above that of healthy SF while remaining fully compatible with high-shear joint motion. At 10 rad/s, G′ exceeded G″ in all three cases (148.6/133.6, 126.4/85.9, and 189.8/118.7 Pa), with tan δ values of 0.89, 0.67, and 0.62 and elasticity values of 52.7%, 59.5%, and 61.5%, indicating meaningful energy storage without excessive rigidity. This parallels an independent finding for another linear HA pair: Porcello et al. [14] reported that doubling HA concentration (Ostenil to Ostenil Plus) produced a similarly large increase in G′ and G″, attributed to HA content—the same concentration-driven improvement seen here from TrHLINE 1%^®^ to TrHLINE 2%^®^.

G′/G″ crossover frequencies within this group are not uniform but vary in a functionally meaningful way: TrHLINE 2%^®^ at approximately 8 rad/s, Hyalubrix^®^ at approximately 3 rad/s, and Arthrum H 2%^®^ at approximately 2 rad/s. Hyalubrix^®^ most closely matches the crossover frequency of healthy SF (~3 rad/s), transitioning to elastic dominance precisely at the onset of walking-speed joint loading. TrHLINE 2%^®^ remains viscous-dominant through low-to-moderate activity and transitions to elastic dominance only under higher mechanical demand. It provides the widest adaptive working window within this group. Arthrum H 2%^®^ crosses over slightly below walking frequency (~2 rad/s) and shares this value numerically with Pronolis^®^ HD 2.5% (Group 3). However, crossover frequency alone does not discriminate functional profiles here. As established in Section 2.2 and Section 2.3, Arthrum H 2%^®^ maintained a continuously rising stress response and a sustained N1 profile reaching approximately 2400 Pa at maximum shear. On the other hand, Pronolis^®^ HD 2.5% exhibited declining stress and a non-monotonic N1, indicating reduced structural persistence under progressive dynamic demand. This divergence confirms that sustained structural coherence under shear—not crossover frequency—is the defining mechanical signature of Group 2.

N1 data further consolidate this classification: TrHLINE 2%^®^ and Arthrum H 2%^®^ rose continuously to approximately 2800 and 2400 Pa at maximum shear, while Hyalubrix^®^ showed a lower but sustained profile throughout. TrHLINE 2%^®^ generated the highest and most continuously sustained N1 values in the dataset despite lower low-shear viscosity than Arthrum H 2%^®^. Such behavior suggests a particularly effective load-responsive behavior under dynamic conditions. Collectively, shear-thinning ratios within the healthy SF range, sustained stress resistance, and progressive N1 development defined a distinct rheological profile not observed in either lubricant-oriented or high-density elastic formulations. Hyalubrix^®^ has demonstrated clinical efficacy in moderate-to-severe glenohumeral OA [32], supporting the relevance of this mechanical profile beyond the knee. Group 2 is hypothesized to be best suited to established KOA (KL II–III) in patients with meaningful functional demand— including active and semi-active individuals—where progressive cartilage loss increases reliance on SF for dynamic load redistribution across the full physiological activity range.

**Group 3—High-density elastic formulations: Biolevox™ HA ONE 2.5% and Pronolis^®^ HD 2.5%.** The rheological profile of these formulations is defined by high initial moduli (G′ 357.4 and 424.0 Pa at 10 rad/s), G′/G″ crossover at approximately 1 and 2 rad/s respectively, and elasticity values (67.9% and 61.3%) exceeding both healthy and OA SF. These properties confer strong immediate elastic resistance under static and low-mobility loading. The limitations of this profile emerge under dynamic demand: both formulations showed declining stress response and non-monotonic N1 at higher shear rates, indicating reduced structural persistence under progressive mechanical loading. Shear-thinning ratios (877.58 and 509.77) far exceeding the healthy SF reference range (60–150) reflect steep viscosity decay under shear. This is consistent with the known concentration- and molecular-weight dependence of HA rheology, whereby denser chain entanglement raises viscosity and moduli but favors an abrupt rather than gradual viscoelastic transition [13,25]. Both formulations are tested at the highest HA concentration in this panel (25 mg/mL; Table 3), and Biolevox™ HA ONE 2.5% carries the highest documented molecular weight (1.8–3.2 MDa; Table 3) among the eight products; molecular weight is not disclosed for Pronolis^®^ HD 2.5%.

Overall, these materials display high initial structural resistance, effective under predominantly static or low-mobility conditions, but limited mechanical persistence, restricting sustained load redistribution under cyclic loading conditions. Available clinical evidence is consistent with this interpretation: benefit with Pronolis^®^ HD 2.5% has been reported in a predominantly older, overweight and functionally restricted KL II–III population (91.6% of patients) [33], in whom joint loading is largely static and dynamic structural persistence is not the primary mechanical requirement. Thus, Group 3 formulations address the same severity range as Group 2 (KL II–III) but are hypothesized to be more appropriate for sedentary or mobility-restricted patients who require acute, high-cushioning protection rather than sustained mechanical support across a broad activity spectrum.

**Towards indication-oriented product selection.** The three functional profiles identified in this study would carry distinct mechanical implications for the osteoarthritic joint. Formulations below the OA SF rheological floor—Hyalgan Bio^®^ and Suplasyn^®^—offer limited mechanical restoration by any parameter examined. From TrHLINE 1%^®^ onward, all tested formulations exceeded OA SF thresholds to varying extents, indicating rheological characteristics consistent with potential mechanical support in osteoarthritic joints. The rheological stratification reported here—lubricant-oriented, balanced viscoelastic, and high-density elastic—suggests that product selection aligned with patient functional demand might be clinically relevant, with formulations differing not only in the magnitude but in the nature of the mechanical support they provide.

These are in vitro observations performed on undiluted products under standardized conditions, enabling robust side-by-side comparison of intrinsic rheological behavior. In situ dilution, enzymatic degradation, oxidative cleavage, and synovial clearance—processes not captured here—might modify these profiles in the joint environment [10,23,34,35,36]. Measurements were performed at 25 °C rather than at joint temperature (~33–35 °C); this is standard practice in HA viscosupplement rheological characterization, including in Nicholls et al. [23], the only directly comparable multi-product benchmarking study in this clinical space, and is adopted here to ensure comparability across products, instruments, and laboratories without the additional variability introduced by physiological-temperature control. Additionally, stress and N1 were recorded under a single ascending shear rate ramp; the resulting decline in these parameters at high shear for Biolevox™ HA ONE 2.5% and Pronolis^®^ HD 2.5% is interpreted as reduced structural persistence rather than as demonstrated irreversible degradation, since descending ramps, recovery tests, or thixotropic measurements—which would be needed to distinguish reversible shear-thinning from irreversible structural damage—were not performed here and represent a natural extension of this benchmarking framework. The mechanical profiles identified herein establish a rheological baseline for comparative product assessment and hypothesis generation. Their clinical relevance, however, requires validation through controlled studies correlating rheological characteristics with patient-reported and functional outcomes across OA severity grades and activity levels.

## 3. Conclusions

This study demonstrates that commercial linear HA hydrogels are not mechanically interchangeable and cannot be classified by any single parameter. Multi-parametric rheological benchmarking resolved three functional profiles. Lubricant-oriented formulations (Hyalgan Bio^®^, Suplasyn^®^, TrHLINE 1%^®^) showed the lowest mechanical complexity, with TrHLINE 1%^®^ uniquely matching healthy SF viscosity among this group. Balanced viscoelastic formulations (TrHLINE 2%^®^, Hyalubrix^®^, Arthrum H 2%^®^), distinguished by sustained N1, stable stress response, and crossover behavior within the physiological frequency range, most closely reproduce the dynamic mechanical identity of healthy SF. High-density elastic formulations (Biolevox™ HA ONE 2.5%, Pronolis^®^ HD 2.5%) are characterized by early elastic dominance and reduced structural persistence under dynamic loading. These findings support a hypothesized indication framework in which rheological profile, OA severity, and patient functional demand are treated as complementary—though not yet clinically validated—determinants of product selection, pending controlled clinical studies linking rheological behavior to patient outcomes.

## 4. Materials and Methods

### 4.1. Materials and Chemicals

Eight different linear HA commercial injectable hydrogels designed as intra-articular fillers were compared in this study: TrHLINE 1%^®^, TrHLINE 2%^®^, Suplasyn^®^, Hyalgan Bio^®^, Hyalubrix^®^, Arthrum H 2%^®^, Pronolis^®^ HD 2.5% and Biolevox^TM^ HA ONE 2.5%.

All competitor products were purchased independently through standard Spanish community pharmacy channels, matching the acquisition route available to prescribers and patients, with no pre-selection based on anticipated rheological performance. Rheological measurements were performed by i+Med’s analytical department, organizationally independent from the authors responsible for study design and data interpretation, following the standardized protocol described above uniformly across all eight products. Their commercial information is summarized in Table 3. Molecular weight and pH values reported in Table 3 were obtained from each product’s manufacturer instructions for use (IFU) or in commercial information by the manufacturer; these values were not independently verified by the authors.

### 4.2. Rheological Characterization

An AR550 rheometer (TA Instruments, New Castle, DE, USA) was used for the rheological characterization. All measurements were performed at 25 °C using a cone-plate geometry (40 mm diameter, 4° cone angle). The flow ramp was conducted as a continuous ascending ramp from 0.01 to 1000 s^−1^, as done previously by our group [28]. Shear viscosity (η), mechanical shear stress (σ), and first normal stress difference (N1) were recorded simultaneously from each flow ramp experiment and output directly by the instrument software. Edge conditions were visually monitored throughout each ramp, and no sample ejection or edge fracture was observed within the reported shear rate range. All measurements were performed in duplicate as instrumental quality-control checks; both runs showed close agreement, confirming measurement reproducibility, and representative values are reported. Then, the viscosity–shear rate ratio was determined using Equation (1):

(1) $$\eta :\gamma \;ratio=\frac{{\eta \;at\;\gamma \;1\;s}^{-1}\;}{{\eta \;at\;\gamma \;1000\;s}^{-1}}$$

In addition, the elastic (G′) and viscous (G″) moduli were calculated as a function of frequency from 0.1 to 63 rad/s, fixing the strain at 1% [28]. A strain amplitude sweep was performed for each of the eight formulations prior to the frequency sweep; G′ and G″ remained essentially unchanged between 1% and 10% strain in every case, with deviation from the linear plateau observed only at or above 100% strain (Supplementary Table S3). Finally, complex modulus (G*), loss factor (tan δ) and elasticity (%) were calculated using the following equations.

(2) $$Complex\;modulus\;(G^{*})=\sqrt{{\left(G^{\prime }\right)}^{2}+{\left(G^{\prime \prime }\right)}^{2}}$$

(3) $$Loss\;factor\;(\tan\delta )=\frac{G^{\prime \prime }}{G^{\prime }}$$

(4) $$Elasticity\;\left(\%\right)=\frac{G^{\prime }}{G^{\prime }+G^{\prime \prime }}\cdot 100$$

This descriptor is algebraically equivalent to the loss factor, Elasticity % = 100/(1 + tan δ), and re-expresses the same viscous–elastic balance on an intuitive 0–100% scale; the metric follows established usage in HA rheological characterization of crosslinked dermal fillers, where percent elasticity—calculated by the same formula—has been reported as a standard comparative descriptor across formulations [29].

### 4.3. Statistical Analysis

All rheological measurements were performed in duplicate (*n* = 2) as instrumental quality-control replicates (Section 2.2). Given this sample size, only the arithmetic mean was calculated as a summary descriptor, together with the standard error of the mean (SEM). The representative values reported in Section 2 (Table 1, Figure 1, Figure 2, Figure 3, Figure 4 and Figure 5) correspond to one of the two replicate measurements per product, confirmed to closely agree with the second replicate (Supplementary Table S1). Complete duplicate datasets, including mean ± SEM values and individual replicate curves for all eight tested products, are provided in the Supplementary Materials (Tables S1–S3, Figures S1–S5).

## Figures and Tables

**Figure 1:**
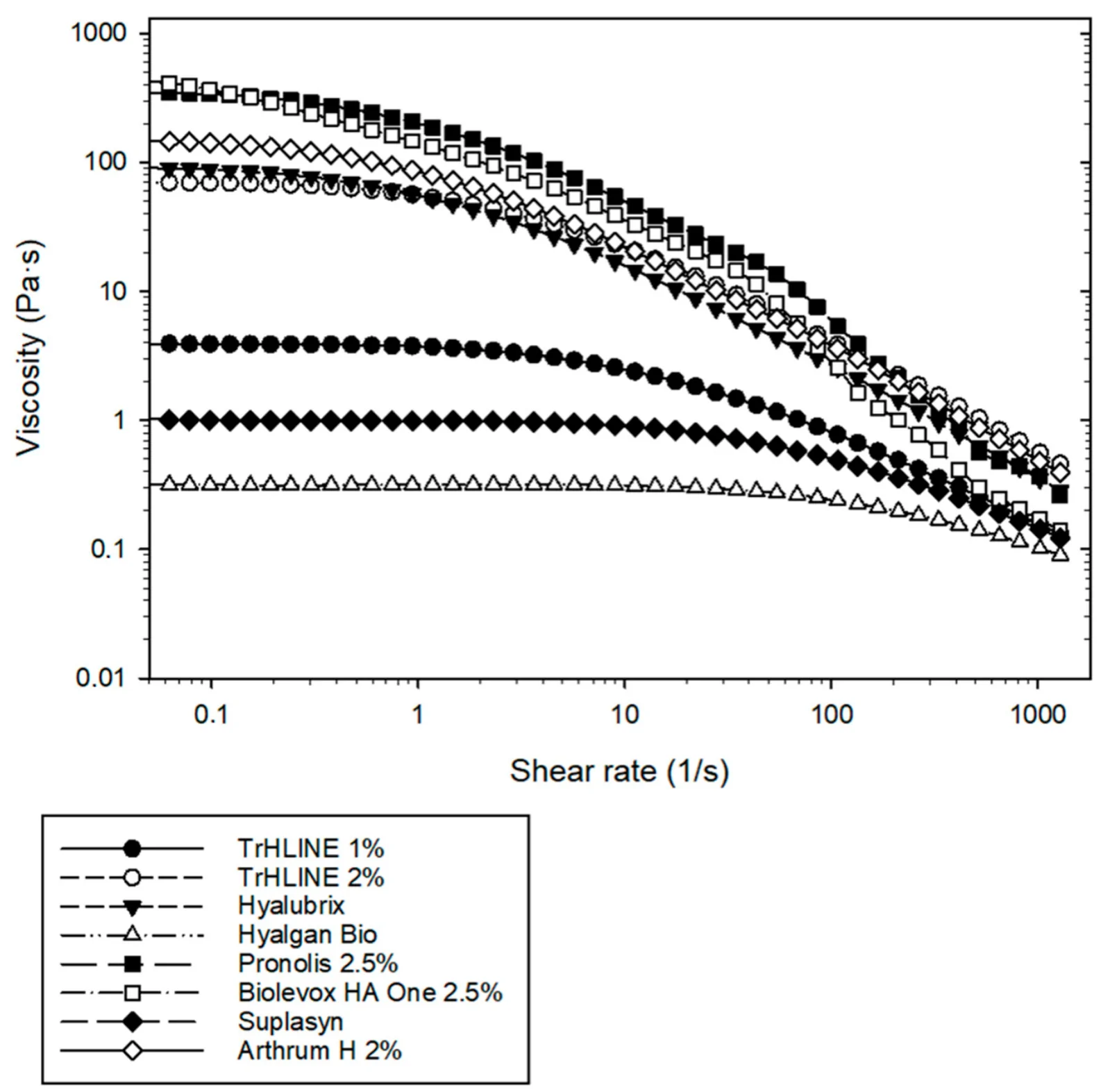
Shear viscosity (Pa·s) vs. shear rate (s^−1^) for the eight hyaluronic acid formulations, illustrating their shear-thinning behavior and differences in the low- and high-shear viscosity range.

**Figure 2:**
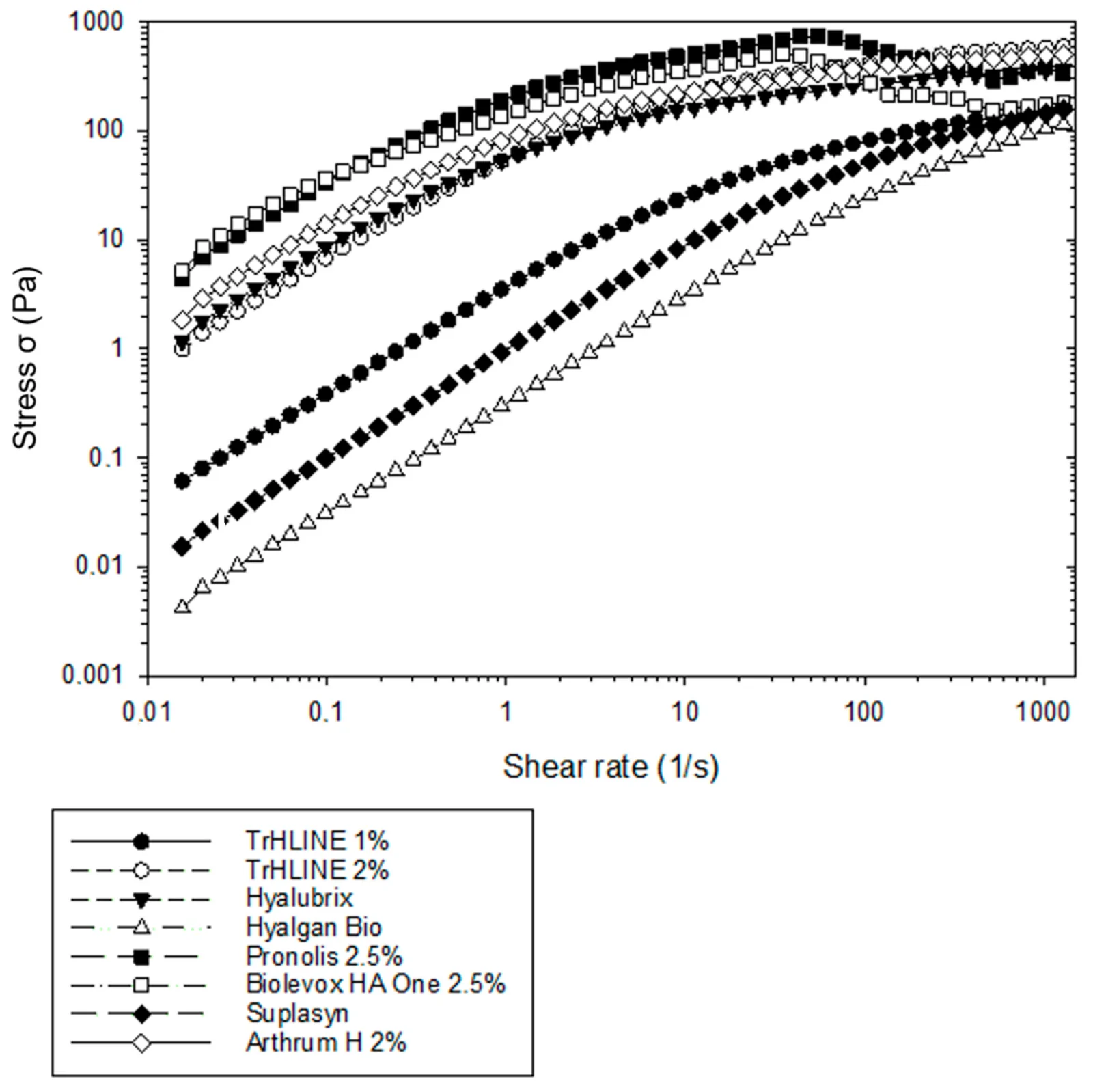
Stress (σ, Pa) as a function of shear rate (s^−1^), showing differences in mechanical resistance and structural stability under increasing shear conditions.

**Figure 3:**
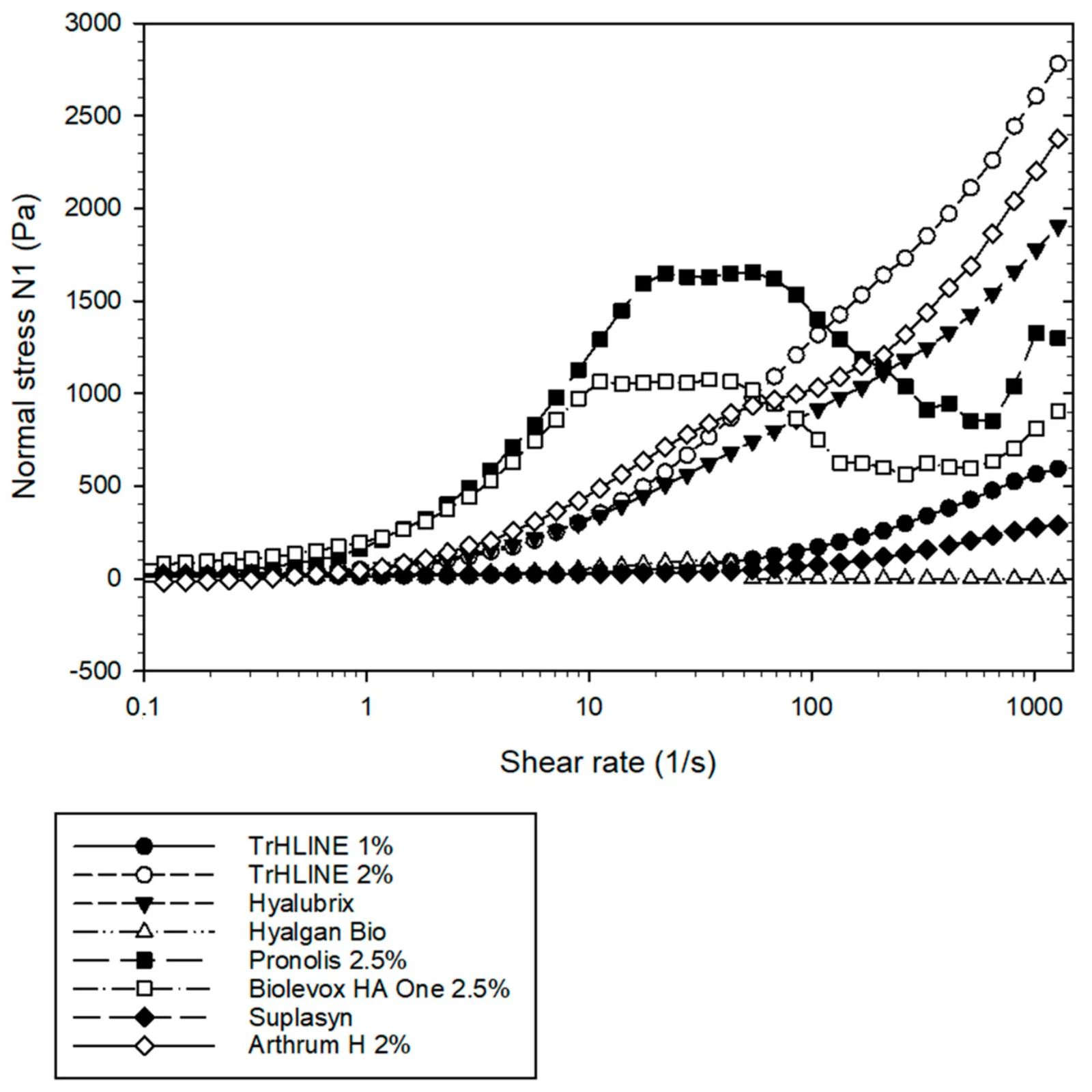
First normal stress difference (N1, Pa) versus shear rate (s^−1^), reflecting the load-dependent elastic response and the capacity for mechanical load redistribution.

**Figure 4:**
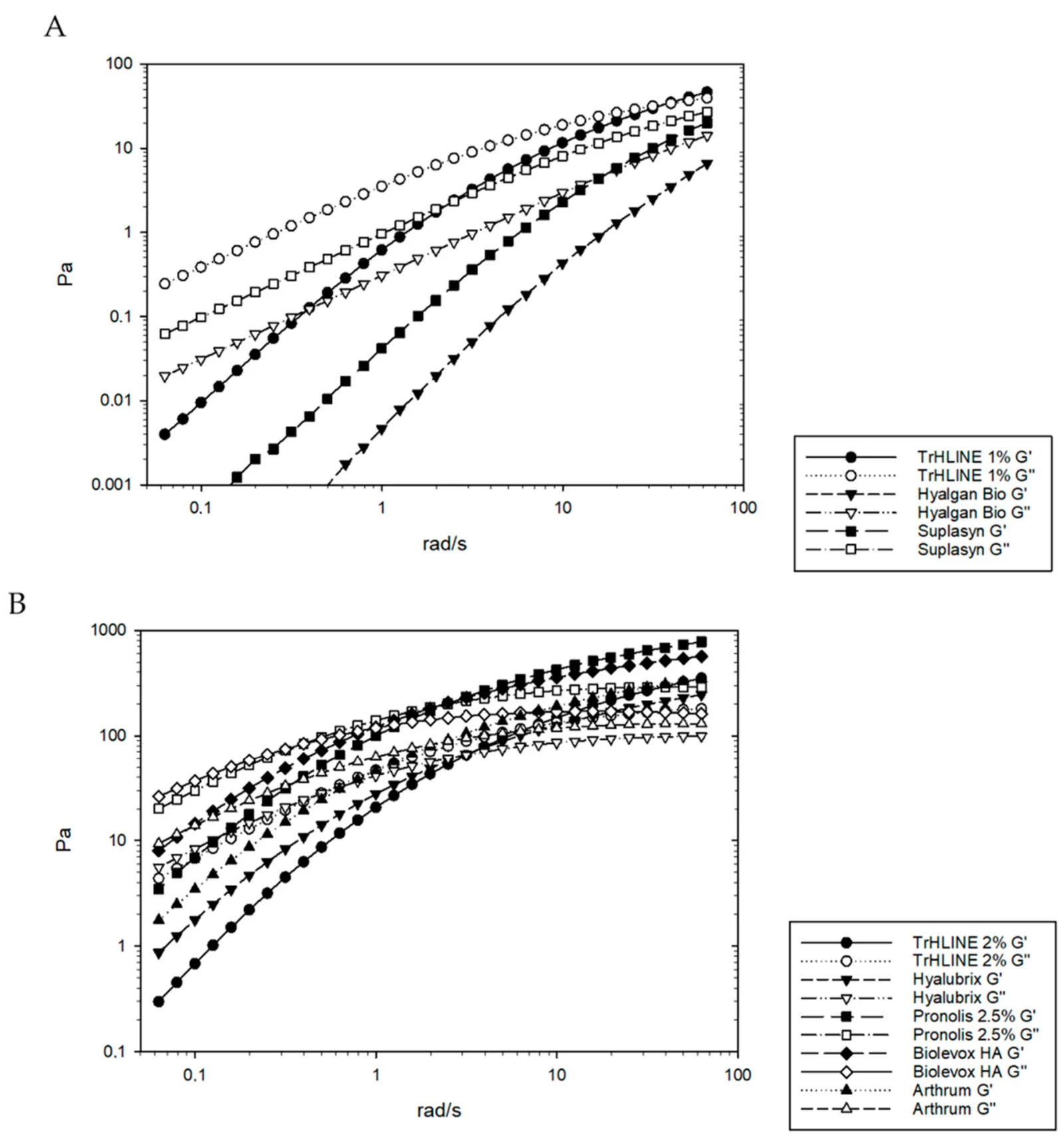
Storage modulus (G′) and loss modulus (G″) as a function of angular frequency (rad/s), indicating viscoelastic behavior and crossover points between elastic and viscous dominance. (**A**) corresponds to low HA% products, (**B**) corresponds to >1.5% HA products.

**Figure 5:**
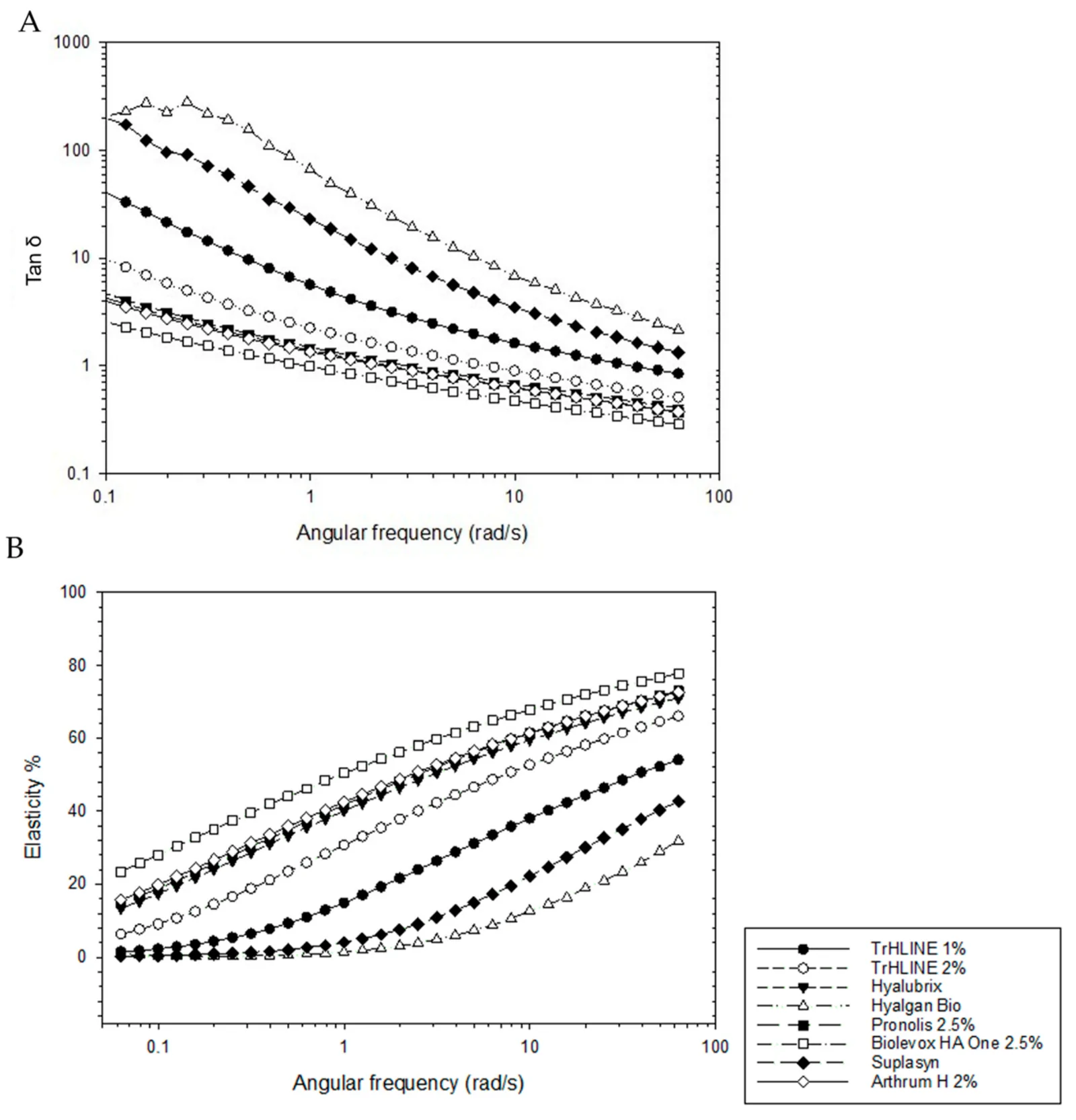
(**A**) Loss factor (tan δ) and (**B**) elasticity (%) as a function of angular frequency, describing the balance between viscous and elastic contributions to material behavior.

**Table 1:** Steady-shear viscosity and oscillatory viscoelastic parameters of HA viscosupplements. Values shown are representative of one of two independent instrumental measurements per product (Section 4.3); the second replicate measurement, which showed close agreement with the reported value, is available in the Supplementary Materials. G′, G″, tan δ and elasticity % are given at 10 rad/s. Derived parameters (η ratio, tan δ, elasticity %) were calculated from unrounded instrument values.

	TrHLINE 1%^®^	TrHLINE 2%^®^	Hyalubrix^®^	Hyalgan Bio^®^	Pronolis^®^ HD 2.5%	Biolevox™ HA ONE 2.5%	Suplasyn^®^	Arthrum H 2%^®^
η (Pa·s) 0.1 s^−1^	3.90	68.94	86.89	0.31	331.12	342.47	0.99	138.86
η (Pa·s) 1 s^−1^	3.70	53.37	52.47	0.31	187.40	148.83	0.99	79.14
η (Pa·s) 1000 s^−1^	0.15	0.56	0.35	0.10	0.36	0.17	0.14	0.48
η:γ ratio	23.84	94.88	148.81	3.12	509.77	877.58	6.94	164.82
G′ (Pa)	11.61	148.64	126.36	0.43	423.96	357.38	2.29	189.81
G″ (Pa)	18.88	133.58	85.91	2.95	267.10	169.28	8.05	118.70
G* (Pa)	22.16	199.84	152.80	2.98	501.08	395.45	8.37	223.87
Moduli Crossover	~30 rad/s	~8 rad/s	~3 rad/s	-	~2 rad/s	~1 rad/s	-	~2 rad/s
tan δ	1.62	0.89	0.67	6.84	0.63	0.47	3.50	0.62
Elasticity %	38.07	52.66	59.52	12.75	61.34	67.85	22.18	61.52

**Table 2:** Rheological and molecular characteristics of healthy and osteoarthritic synovial fluid (SF). Values are representative reference ranges reported in the literature; where no numerical value is available, this is indicated accordingly.

Parameter	Healthy SF	Osteoarthritic SF
HA concentration (mg/mL)	1–4	1–2
HA molecular weight (MDa)	6–8	0.5–3.5
η (Pa·s), zero-shear	~3	0.1–1
η:γ ratio	60–150	5–40
G′ (Pa)	~23	~7
tan δ	~0.30	not reported numerically in the literature
G′/G″ crossover	~3 rad/s	not reported numerically in the literature
References	[9,12,13,14,15,16,17,18]	[9,12,13,18,30]

**Table 3:** Commercial information on HA intra-articular fillers. Molecular weight and pH values are as reported in each product’s instructions for use (IFU); in the case of TrHLINE molecular weight corresponds to the HA used for manufacturing; a dash (—) indicates the parameter is not specified.

Product Name	HA Concentration (mg/mL)	Volume per Syringe (mL)	HA Origin	Molecular Weight	pH
TrHLINE 1%^®^	10 mg/mL	2 mL	Fermentation	2.0 MDa	Physiological
TrHLINE 2%^®^	20 mg/mL	2 mL	Fermentation	2.0 MDa	Physiological
Hyalgan Bio^®^	10 mg/mL	2 mL	Natural HA (Hyalectin^®^ from rooster combs)	0.5–0.75 MDa	6.8–7.5
Suplasyn^®^	10 mg/mL	2 mL (or 6 mL Suplasyn^®^ 1-shot)	Fermentation	—	—
Hyalubrix^®^	15 mg/mL	2 mL	Fermentation	1.5–2.0 MDa	Physiological
Arthrum H 2%^®^	20 mg/mL	2 mL	Fermentation	2.8 MDa	—
Pronolis^®^ HD 2.5%	25 mg/mL	4.8 mL	Fermentation	—	6.8–7.4
Biolevox™ HA ONE 2.5%	25 mg/mL	4.8 mL	Fermentation	1.8–3.2 MDa	6.8–7.4

## Data Availability

All data generated and analyzed during this study are included in this published article and its Supplementary Materials. This includes the data from both technical duplicate measurements performed for all eight formulations, the corresponding summary statistics, Table 2, Figure 1, Figure 2, Figure 3, Figure 4 and Figure 5, Supplementary Tables S1–S3, and Supplementary Figures S1–S5. Raw instrumental files and complete instrument-generated measurement records are available from the corresponding author upon reasonable request.

## Supplementary Materials

The following supporting information can be downloaded from https://www.mdpi.com/article/10.3390/gels12100882/s1. Supplementary Table S1. Steady-shear viscosity and oscillatory viscoelastic parameters of HA viscosupplements. Values shown are the second replicate measurement, which showed close agreement with the reported value in the main text (Table 1). G′, G″, tan δ and Elasticity % are given at 10 rad/s. Derived parameters (η ratio, tan δ, elasticity %) were calculated from unrounded instrument values. Supplementary Table S2. Steady-shear viscosity and oscillatory viscoelastic parameters of HA viscosupplements, expressed as mean ± standard error of the mean (SEM) of the two independent instrumental replicates (n = 2; Section 2.3), one of which corresponds to the representative value reported in the main text (Table 2). G′, G″, tan δ and Elasticity % are given at 10 rad/s. Derived parameters (η:γ ratio, tan δ, elasticity %, G*) were calculated independently for each replicate from unrounded instrument values before averaging. A dash (–) indicates that no G′/G″ crossover was observed within the explored frequency range for either replicate. Supplementary Table S3. Storage and loss moduli (G′, G″) of HA viscosupplements from oscillatory amplitude sweeps, given at 1%, 10% and 100% strain. Values shown are the representative replicate measurement reported in the main text (Table 1). Supplementary Figure S1. Shear viscosity (Pa·s) vs Shear rate (s^−1^) for the eight hyaluronic acid formulations, illustrating their shear-thinning behavior and differences in low- and high-shear viscosity range. Supplementary Figure S2. Stress (σ, Pa) as a function of shear rate (s^−1^), showing differences in mechanical resistance and structural stability under increasing shear conditions. Supplementary Figure S3. First normal stress difference (N1, Pa) versus shear rate (s^−1^), reflecting the load-dependent elastic response and capacity for mechanical load redistribution. Supplementary Figure S4. Storage modulus (G′) and loss modulus (G″) as a function of angular frequency (rad/s), indicating viscoelastic behavior and crossover points between elastic and viscous dominance. (A) corresponds to low HA% products, (B) corresponds to >1.5% HA products. Supplementary Figure S5. (A) Loss factor (tan δ) and (B) elasticity (%) as a function of angular frequency, describing the balance between viscous and elastic contributions to material behavior.

## Abbreviations

The following abbreviations are used in this manuscript:

OA	Osteoarthritis
KOA	Knee osteoarthritis
SF	Synovial Fluid
HA	Hyaluronic Acid
KL	Kellgren–Lawrence
ROS	Reactive Oxygen Species
N_1_	First normal stress difference
G′	Storage modulus
G″	Loss modulus
tan δ	Loss factor
