# Supplementary material for: Comparative Rheological Characterization of Hyaluronic Acid Viscosupplements for Synovial Fluid Restoration in Osteoarthritis and Knee Biomechanics
Source: Gels. 2026 Sep 30;12(10):882. doi: 10.3390/gels12100882 (PMC13704416; doi:10.3390/gels12100882)
Supplement: Supplementary file 1 [file gels-12-00882-s001.zip › gels-4509587-supplementary.pdf]

Supplementary Data.

Supplementary Table S1. Steady-shear viscosity and oscillatory viscoelastic parameters of HA viscosupplements. Values shown are the second replicate measurement, which showed close agreement with the reported value in the main text (Table 1). G', G'', tan δ and Elasticity % are given at 10 rad/s. Derived parameters (η ratio, tan δ, elasticity %) were calculated from unrounded instrument values.

	TrHLINE 1%®	TrHLINE 2%®	Hyalubrix ®	Hyalgan Bio®	Pronolis® HD 2.5%	Biolevox ™ HA ONE 2.5%	Suplasyn ®	Arthrum H 2%®
η (Pa·s) 0.1 s^{-1}	3.22	52.23	89.22	0.32	275.71	382.14	1.05	149.39
η (Pa·s) 1 s^{-1}	3.14	42.5	56.07	0.33	165.54	149.78	1.04	87.9
η (Pa·s) 1000 s^{-1}	0.15	0.47	0.35	0.1	0.28	0.15	0.14	0.49
η:γ ratio	20.8	90.47	158.61	3.18	601.86	967.11	7.22	178.23
G' (Pa)	9.83	117.87	126.6	0.43	377.11	367.83	2.31	190.14
G'' (Pa)	16.98	109.51	85.97	2.97	234.23	175.25	8.08	118.76
G* (Pa)	19.62	160.89	153.03	3.0	443.93	407.45	8.41	224.18
Moduli Crossover	~43 rad/s	~8 rad/s	~3 rad/s	-	~2 rad/s	~1 rad/s	-	~2 rad/s
tan δ	1.73	0.93	0.68	6.82	0.62	0.48	3.5	0.62
Elasticity %	36.66	51.84	59.56	12.78	61.69	67.73	22.23	61.55

Supplementary Table S2. Steady-shear viscosity and oscillatory viscoelastic parameters of HA viscosupplements, expressed as mean ± standard error of the mean (SEM) of the two independent instrumental replicates (n = 2; Section 2.3), one of which corresponds to the representative value reported in the main text (Table 2). G′, G″, tan δ and Elasticity % are given at 10 rad/s. Derived parameters (η:γ ratio, tan δ, elasticity %, G*) were calculated independently for each replicate from unrounded instrument values before averaging. A dash (–) indicates that no G′/G″ crossover was observed within the explored frequency range for either replicate.

	TrHLINE 1%®	TrHLINE 2%®	Hyalubrix ®	Hyalgan Bio®	Pronolis® HD 2.5%	Biolevox ™ HA ONE 2.5%	Suplasyn ®	Arthrum H 2%®
η (Pa·s) 0.1 s^{-1}	3.56 ± 0.34	60.59 ± 8.35	88.05 ± 1.16	0.32 ± 0.01	303.42 ± 27.70	362.30 ± 19.83	1.02 ± 0.03	144.12 ± 5.26
η (Pa·s) 1 s^{-1}	3.42 ± 0.28	47.93 ± 5.44	54.27 ± 1.80	0.32 ± 0.01	176.47 ± 10.93	149.31 ± 0.48	1.01 ± 0.02	83.52 ± 4.38
η (Pa·s) 1000 s^{-1}	0.151 ± 0.001	0.515 ± 0.045	0.352 ± 0.002	0.102 ± 0.002	0.318 ± 0.042	0.162 ± 0.008	0.142 ± 0.002	0.487 ± 0.007

η:γ ratio	22.32 ± 1.52	92.68 ± 2.20	153.71 ± 4.90	3.15 ± 0.03	555.82 ± 46.05	922.34 ± 44.76	7.08 ± 0.14	171.53 ± 6.71
G' (Pa)	10.72 ± 0.89	133.25 ± 15.39	126.48 ± 0.12	0.43 ± 0.00	400.53 ± 23.43	362.60 ± 5.22	2.30 ± 0.01	189.98 ± 0.17
G'' (Pa)	17.93 ± 0.95	121.55 ± 12.03	85.94 ± 0.03	2.96 ± 0.01	250.66 ± 16.44	172.27 ± 2.99	8.07 ± 0.02	118.73 ± 0.03
G* (Pa)	20.89 ± 1.27	180.37 ± 19.47	152.92 ± 0.12	2.99 ± 0.01	472.51 ± 28.57	401.45 ± 6.00	8.39 ± 0.02	224.03 ± 0.16
Moduli Crossover	36.5 ± 6.5	8.0 ± 0.0	3.0 ± 0.0	–	2.2 ± 0.2	1.0 ± 0.0	–	2.1 ± 0.1
tan δ	1.67 ± 0.05	0.91 ± 0.02	0.67 ± 0.00	6.83 ± 0.01	0.63 ± 0.00	0.47 ± 0.00	3.50 ± 0.00	0.62 ± 0.00
Elasticity %	37.37 ± 0.70	52.25 ± 0.41	59.54 ± 0.02	12.77 ± 0.02	61.51 ± 0.17	67.79 ± 0.06	22.21 ± 0.03	61.54 ± 0.02

Supplementary Table S3. Storage and loss moduli (G′, G″) of HA viscosupplements from oscillatory amplitude sweeps, given at 1%, 10% and 100% strain. Values shown are the representative replicate measurement reported in the main text (Table 1).

	G'(1%)	G'(10%)	G'(100%)	G''(1%)	G''(10%)	G''(100%)
TrHLINE 2%®	98.95	98.87	83.78	97.03	97.01	96.04
TrHLINE 1%®	5.87	5.88	5.21	12.69	12.75	12.41
Pronolis HD 2.5%	316.89	316.50	265.42	219.52	219.31	209.46
Biolevox™ HA ONE 2.5%	332.89	328.58	290.00	178.59	182.68	195.97
Hyalubrix®	101.08	101.11	84.81	78.63	78.50	75.14
Hyalgan Bio®	0.18	0.19	0.17	1.96	1.96	1.96
Arthrum H 2%®	150.06	150.76	125.89	110.25	110.17	105.14
Suplasyn®	1.21	1.23	1.14	5.76	5.72	5.69

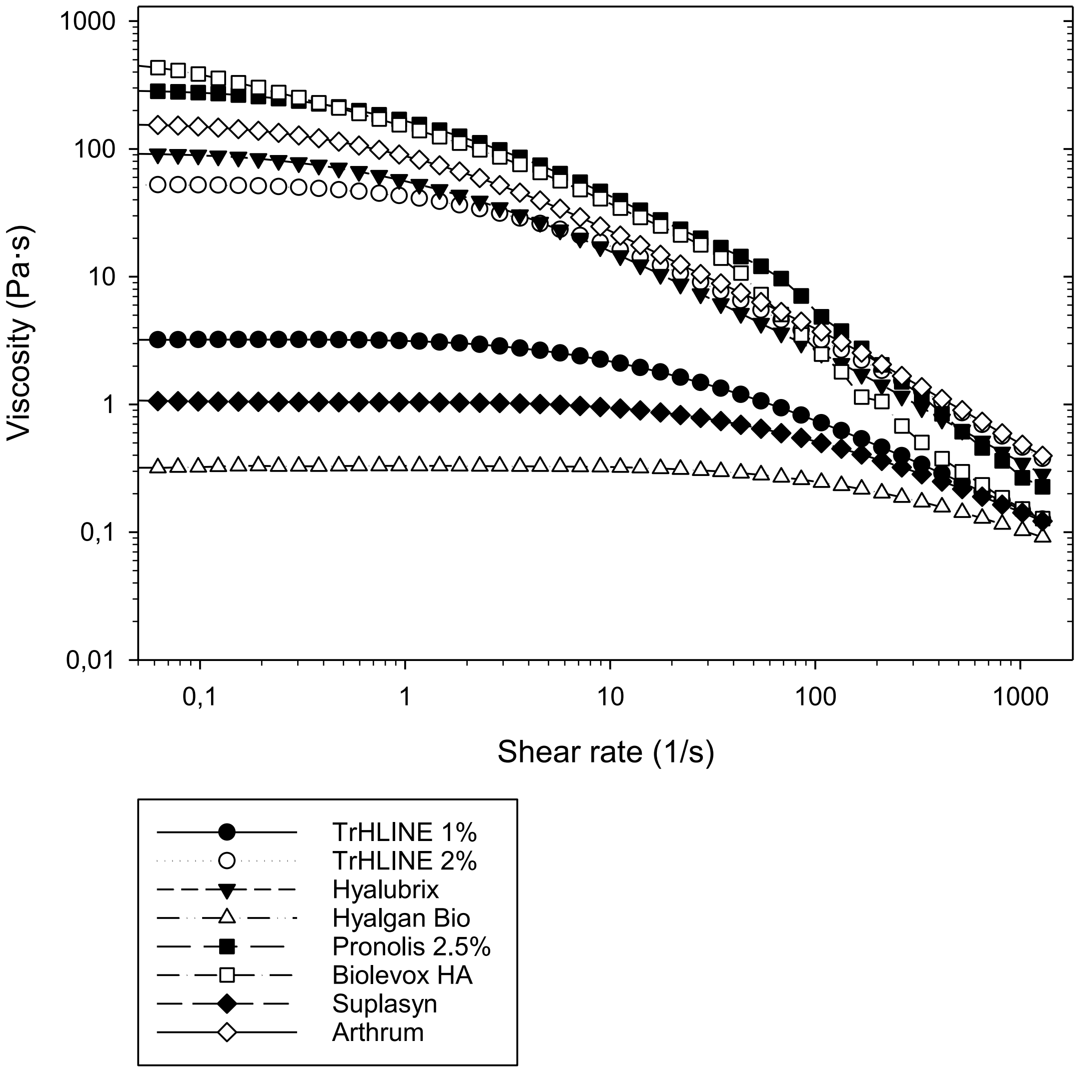

Supplementary Figure S1. Shear viscosity (Pa·s) vs Shear rate (s^{-1}) for the eight hyaluronic acid formulations, illustrating their shear-thinning behavior and differences in low- and high-shear viscosity range.

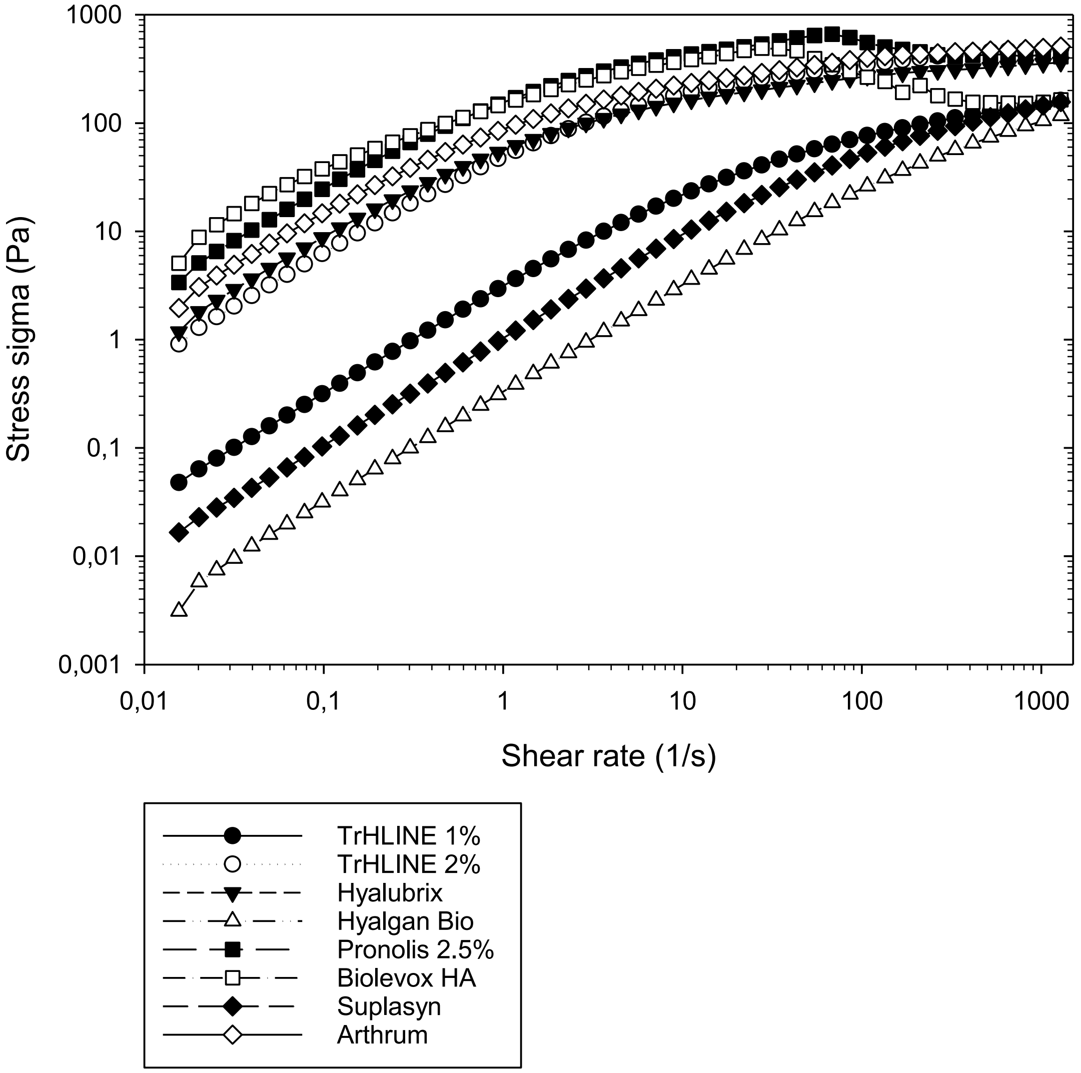

Supplementary Figure S2. Stress (σ, Pa) as a function of shear rate (s^{-1}), showing differences in mechanical resistance and structural stability under increasing shear conditions.

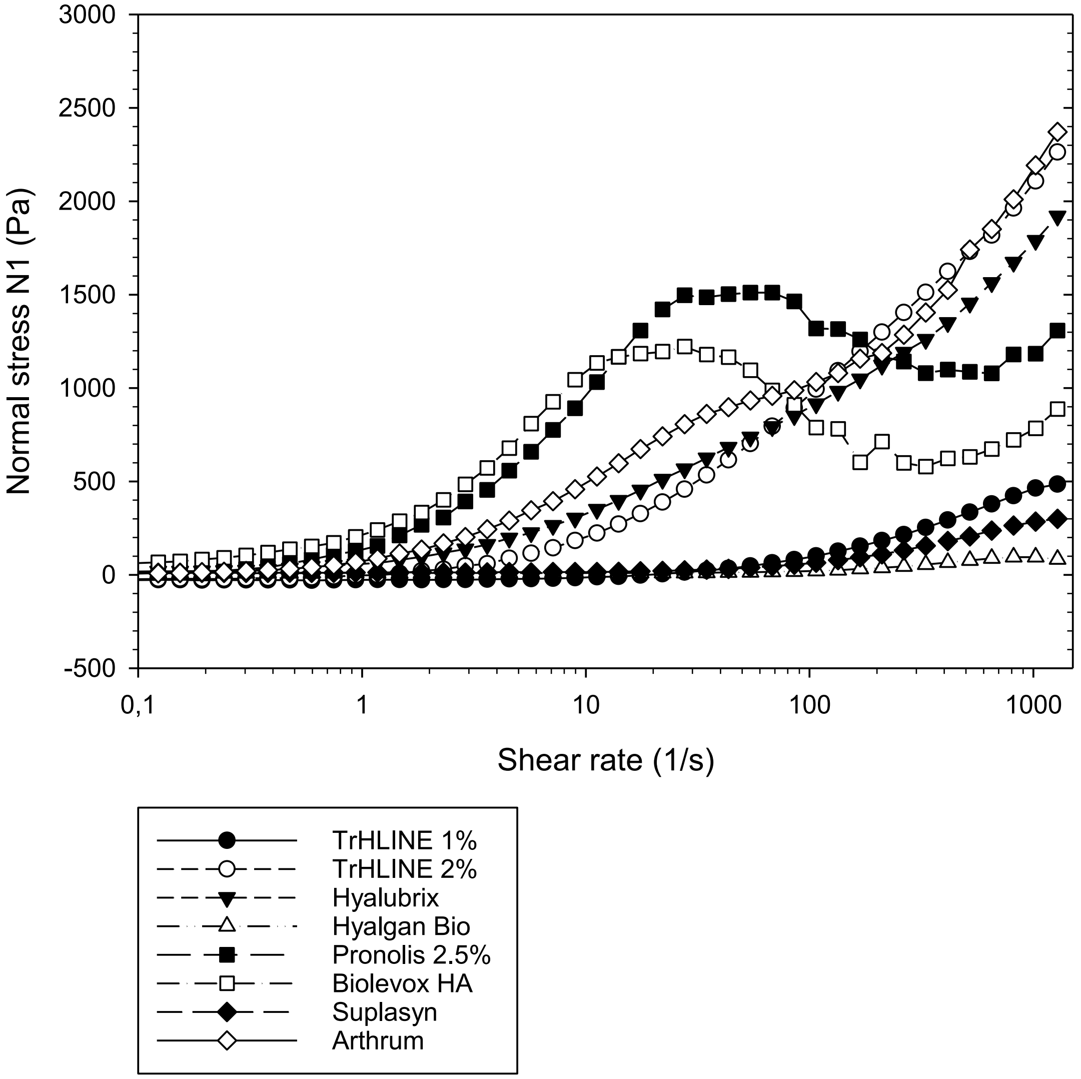

Supplementary Figure S3. First normal stress difference (N1, Pa) versus shear rate (s^{-1}), reflecting the load-dependent elastic response and capacity for mechanical load redistribution.

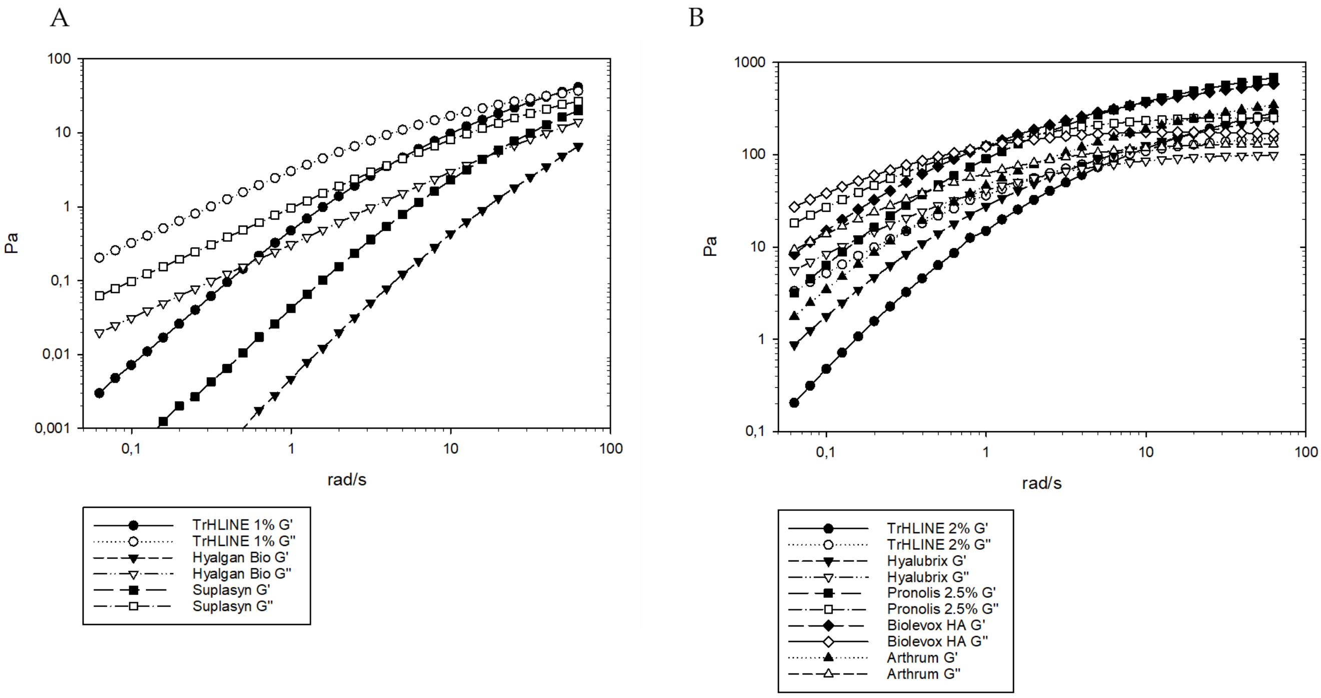

Supplementary Figure S4. Storage modulus (G′) and loss modulus (G″) as a function of angular frequency (rad/s), indicating viscoelastic behavior and crossover points between elastic and viscous dominance. A) corresponds to low HA% products, B) corresponds to >1.5% HA products.

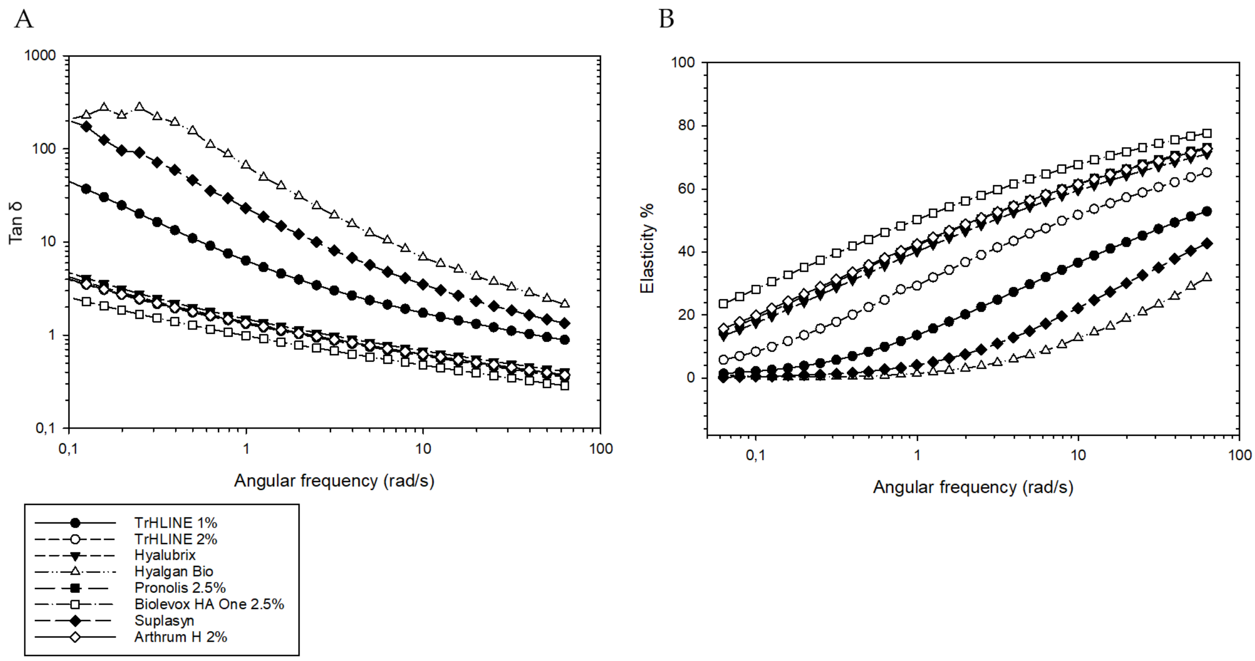

Supplementary Figure S5. (A) Loss factor (tan δ) and (B) elasticity (%) as a function of angular frequency, describing the balance between viscous and elastic contributions to material behavior.
